# The JNK- and AKT/GSK3β- Signaling Pathways Converge to Regulate Puma Induction and Neuronal Apoptosis Induced by Trophic Factor Deprivation

**DOI:** 10.1371/journal.pone.0046885

**Published:** 2012-10-03

**Authors:** Kristin K. Ambacher, Kristen B. Pitzul, Meera Karajgikar, Alison Hamilton, Stephen S. Ferguson, Sean P. Cregan

**Affiliations:** Robarts Research Institute and Department of Physiology and Pharmacology, University of Western Ontario, London, Ontario, Canada; University of Louisville, United States of America

## Abstract

The AKT, GSK3 and JNK family kinases have been implicated in neuronal apoptosis associated with neuronal development and several neurodegenerative conditions. However, the mechanisms by which these kinase pathways regulate apoptosis remain unclear. In this study we have investigated the role of these kinases in neuronal cell death using an established model of trophic factor deprivation induced apoptosis in cerebellar granule neurons. BCL-2 family proteins are known to be central regulators of apoptosis and we have determined that the pro-apoptotic family member Puma is transcriptionally up-regulated in trophic factor deprived neurons and that Puma induction is required for apoptosis *in vitro* and *in vivo*. Importantly, we demonstrate that Puma induction is dependent on both JNK activation and AKT inactivation. AKT is known to regulate a number of downstream pathways, however we have determined that PI3K-AKT inactivation induces Puma expression through a GSK3β-dependent mechanism. Finally we demonstrate that the JNK and AKT/GSK3β pathways converge to regulate FoxO3a-mediated transcriptional activation of Puma. In summary we have identified a novel and critical link between the AKT, GSK3β and JNK kinases and the regulation of Puma induction and suggest that this may be pivotal to the regulation of neuronal apoptosis in neurodegenerative conditions.

## Introduction

Apoptosis is a form of programmed cell death that is required in many physiological processes such as embryogenesis, cell turnover and response to pathogens. On the other hand aberrant apoptosis has been implicated in several neurodegenerative conditions including Parkinson’s disease, Huntington’s disease and Alzheimer’s disease as well as acute injuries such as stroke and spinal cord injury [1]–[5]. Therefore, understanding the upstream signaling pathways that regulate apoptosis in neurons is crucial for the development of treatments for these devastating neurological conditions.

Kinase signaling pathways play a key role in signal transduction in all cellular processes including apoptosis. Three kinase pathways in particular are important for apoptotic signaling in neurons: the c-Jun N-terminal kinase (JNK) pathway, the glycogen synthase kinase-3 (GSK3), and the protein kinase B (AKT) pathway (reviewed in [6]–[8]). The JNK pathway is pro-apoptotic and JNK itself is known to be activated in several models of neuronal apoptosis including excitotoxicity, trophic factor withdrawal and ischemia (reviewed in [9]). Furthermore, inhibition of JNK signaling using genetic and pharmacological approaches has been shown to protect neurons against several different apoptotic stimuli [10]–[13]. Similarly, GSK3β has been found to play a pro-apoptotic role in several models of neuronal cell death including serum deprivation, DNA damage and Aβ induced toxicity [14]–[16]. Additionally, while inhibition of GSK3 promotes cell survival, overexpression of active GSK3β has been shown to promote neuronal apoptosis [17]. In contrast to the JNK and GSK3 pathways, AKT serves as a pro-survival signaling pathway (reviewed in [6]) and inactivation of AKT signaling has been implicated in many apoptotic paradigms [18]–[20]. The AKT pathway can be activated in neurons by trophic factors such as insulin-like growth factor (IGF-1) and nerve growth factor (NGF) resulting in promotion of cell survival and protection of neuronal cells against apoptotic stimuli [21]–[24]. While the JNK-, GSK3β- and AKT- pathways have been established as key players in neuronal apoptosis, the downstream targets that link these kinases to the apoptotic machinery has not been clearly defined.

The intrinsic pathway of apoptosis is mediated by the Bcl-2 family of proteins. These proteins are subdivided into pro-apoptotic, anti-apoptotic and BH3-only pro-apoptotic members (reviewed in [25]). Previous studies have established Bax as the key pro-apoptotic player in diverse neuronal apoptotic paradigms [26]–[31]. In response to apoptotic stimuli Bax translocates to the mitochondria where it causes outer mitochondrial membrane permeabilization and release of cytochrome-c leading to caspase activation and ultimately cell death [32], [33]. Activation of Bax is thought to be dependent on the third class of Bcl-2 proteins – the BH3-domain-only subclass – which includes proteins such as Bad, Noxa, Bid, Bim, Hrk/DP5, and Puma. These BH3-only proteins are activated through transcriptional and post-translational mechanisms in response to distinct cellular stresses [25]. Due to their pivotal role in regulating Bax activation BH3-only proteins have received significant attention as potential targets of kinase pathways involved in the regulation of neuronal apoptosis.

We have investigated the potential role of JNK, GSK3 and AKT signaling in the regulation of BH3-only proteins in cerebellar granule neurons (CGNs) undergoing apoptosis in response to potassium deprivation. This established model of trophic factor deprivation induced neuronal apoptosis is believed to mimic aspects of synaptic dysfunction common to many neuronal injury and neurodegenerative conditions [34]. Apoptotic cell death in this paradigm has been demonstrated to be Bax-dependent [26] and to involve the JNK, GSK3 and AKT signaling pathways [35]–[37]. Importantly, in the present study we demonstrate that the BH3-only member Puma is essential for trophic factor deprivation induced apoptosis in CGNs and establish that the JNK-, AKT- and GSK3-family kinases converge to regulate the transcriptional induction of Puma and neuronal apoptosis.

## Materials and Methods

### Ethics Statement

This study was performed in strict accordance with the recommendations in the Canadian Council on Animal Care Guidelines. The protocol was approved by the Animal Use Subcommittee of the University of Western Ontario (Permit Number: 2009-099).

### Animals

Mice carrying targeted null mutations for Puma or Bim were generated on a C57BL/6 background in the laboratory of Dr. Andreas Strasser (WEHI, Victoria, Australia). The genotyping of these mice was performed as previously described [38], [39]. In other experiments neurons were derived from CD1 mice obtained from Charles River Laboratories (Point-Claire Quebec).

### Neuronal Cell Cultures

Primary cerebellar granule neurons (CGNs) were extracted from P7 mice brains by enzymatic and mechanical dissociations as previously described [40]. Cells were resuspended in Neurobasal medium containing B27 and N2 supplements, 0.5X Glutamax (Invitrogen Canada, Burlington Ontario) and 25 mM potassium chloride (KCl) and plated at a density of 0.75×10^6^ cells/ml of medium. Apoptosis was induced after 7 days by switching culture media to Neurobasal medium containing 0.5X Glutamax and 5 mM KCl. In indicated studies, pharmacological agents were added to cultures simultaneous to medium change at the following concentrations: SP600125, SB415286, recombinant IGF-1, LY294002 (all from Sigma-Aldrich Canada, Oakville Ontario) and AR-A014418 (Calbiochem Gibbstown, New Jersey).

### Adenoviral and Lentiviral shRNA Constructs

Adenovirus expressing HA-tagged constitutively active (CA) AKT (N-terminus of AKT1 fused to a c-Src myristoylation sequence) was obtained from Vector Biolabs (Philadelphia, PA). The Ad-CA-AKT and Ad-GFP vectors were amplified and titred as previously described and CGNs were infected with adenoviruses on the day of plating as previously described [41]. Lentivirus expressing shRNA directed against FoxO3a (sc-37888-V) and control lentivirus (sc-108080) were purchased from Santa Cruz Biotech. CGNs were transduced with lentiviral particles at the time of plating.

### Cell Death and Survival Assays

Apoptosis of CGNs was assessed by examining nuclear morphology following Hoechst 33342 staining as previously described [42]. Briefly, Hoechst stain (1 µg/mL) was added directly to medium and incubated for 20 minutes at 37°C. Cells were visualized by fluorescence microscopy (IX70; Olympus, Tokyo, Japan) and images were captured from random fields with a CCD camera (Q-imaging, Burnaby, British Columbia, Canada). The fraction of apoptotic nuclei characterized by condensed chromatin and/or apoptotic bodies was scored by a blind observer. A minimum of 500 cells were analyzed per treatment.

### TUNEL Staining

Seven day old mouse pups (P7) were anaesthetized with Xylazine:Ketamine (2∶1) and cardiac perfused with 4% paraformaldehyde. The brains were removed and fixed overnight by immersion in 4% paraformaldehyde and then cryoprotected by immersion in 30% sucrose. Sagittal sections of the cerebellum were cut with a cryostat at 20 µm thickness and mounted onto gelatin coated microscope slides. Every 5^th^ section was stained for apoptotic cells using the FragEL DNA fragmentation Detection Kit (Calbiochem) according to manufacturer’s directions. Stained sections were visualized using a Nikon Labophot-2 microscope and images were captured using Image-Pro Plus software. The number of TUNEL positive cells in the internal granule layer (IGL) of cerebellar lamellae was scored by an observer blinded to the genotype. The mean number of TUNEL positive cells per IGL field was determined from a minimum of 24 images captured from 8 sections per animal.

### Mitotracker Red Staining

Mitochondrial membrane potential was assessed using Mitotracker Red ® stain (Invitrogen Canada Burlington, Ontario) as previously described [42]. Briefly, cells were incubated for 20 minutes with 100 nM Mitotracker Red and staining was visualized by fluorescence microscopy using a standard TRITC filter set. Percentage of cells stained with Mitotracker Red was counted and represents percentage of cells in which mitochondrial membrane potential was maintained. A minimum of 500 cells were analyzed per treatment.

### Caspase 3-like Activity Assay

Neurons were collected in caspase lysis buffer (10 mM Hepes, 1 mM KCl, 1.5 mM MgCl2, 10% glycerol, 5 µg/ml aprotinin, 2 µg/ml leupeptin, 0.1% nonyl phenoxylpolyethoxylethanol (NP-40), 1 µM Dithiothreitol (DTT), and 0.2 mg/ml phenylmethanesulphonylfluoride (PMSF)) and lysed on ice for 20 minutes. Protein was separated by centrifugation and 5 µg per sample was incubated with caspase reaction buffer (1 M Hepes, 1 M DTT, 1 mg/ml sucrose, 1 mg/ml 3[(3-Cholamidopropyl)dimethylammonio]-propanesulfonic acid (Chaps) buffer) and 15 µM AC-DEVD-AFC peptide substrate (Biomol). Fluorescence emitted by cleavage of peptide substrate was measured after 15 and 45 minutes using a Victor3 plate reader (Perkin Elmer) and difference in fluorescence between the two time points is used to represent caspase 3-like activity.

### Quantitative RT-PCR

RNA was isolated using Trizol reagent as per manufacturer’s instructions (Invitrogen Canada, Burlington, Ontario) and 40 ng of total RNA was used in one-step SYBR green reverse transcription PCR (QuantiFast SYBR Green PCR Kit; Qiagen, Toronto, ON). The RT-PCR program was performed on a Chromo4 system (MJ Research, Watertown, MA) and changes in gene expression were calculated using the Δ(ΔC_t_) method; S12 transcript was used for normalization as previously described [30]. Values are reported as fold increase in mRNA levels in treated samples over control samples. Primer sequences are available on request.

### Western Blot Analysis

Neurons were lysed and incubated on ice for 25 minutes in RIPA lysis buffer (150mM sodium chloride, 1% NP-40, 0.5% sodium deoxycholate, 0.1% SDS, 50 mM Tris pH 8.0, 1 mM EDTA, 1 mM DTT, 1∶100 protease inhibitor cocktail, 1∶100 phosphatase inhibitor cocktail (Sigma Aldrich)) and whole-cell protein lysates were recovered by centrifugation. Protein concentration was measured using a BCA assay (Pierce, Rockford, IL) and protein was separated on 10% SDS-PAGE gels and transferred to nitrocellulose membrane (GE Healthcare). Membranes were blocked in TBS-T (10 mM Tris, 150 mM NaCl, 0.05% Tween 20) containing 5% fat-free milk for one hour and then incubated overnight with primary antibody in 3% fat-free milk in TBS-T. Membranes were then washed in TBS-T and then incubated in HRP-conjugated IgG secondary antibodies for one hour. Membranes were washed again in TBS-T and visualized by chemiluminescence according to manufacturer’s instructions (SuperECL; Pierce, Rockford IL). The following primary antibodies were used: Phospho-AKT (4058S; Cell Signaling), Phospho- ATF2 (9225S; Cell Signaling), ATF3 (C19; Santa Cruz Biotechnology), Calnexin (H-70; Santa Cruz Biotechnology), Phospho-c-Jun (2316S; Cell Signaling), Phospho-GSK3β (Ab 2479; Abcam), Bim (AAP-330; Stressgen), FoxO3a (H-144; Santa Cruz Biotech), Phospho(Thr32)-FoxO3a (9464; Cell Signaling) and Puma (3043; Prosci Incorporated).

### Data Analysis

Data are reported as mean and standard error of the mean. The value *n* represents the number of neuron cultures from independent experiments/platings or number of pups of indicated genotype from which independent neuron cultures were prepared involving at least three independent experiments. Differences between groups were determined by ANOVA and *post hoc* Tukey’s test and were considered statistically significant when *p*<0.05.

## Results

### Puma Expression is Induced Following Potassium Withdrawal and is Required for Cell Death

**Figure 1:**
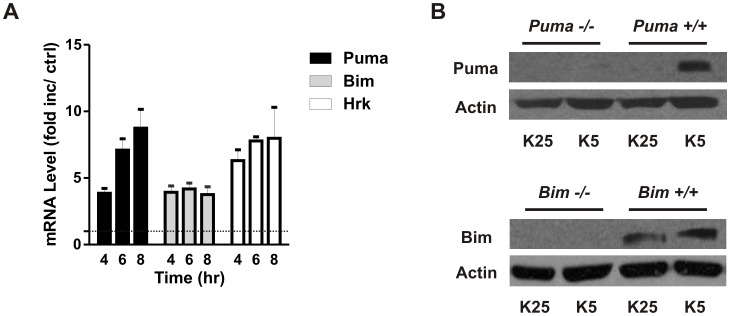
Puma expression is induced by potassium withdrawal in cerebellar granule neurons. After 7 days in culture CGNs were either maintained in media containing 25 mM potassium (K25) or switched to low potassium medium containing 5 mM potassium (K5). **A,** RNA was harvested after 4, 6 and 8 hours and Puma, Bim and Hrk expression was analyzed by qRT-PCR. Expression was normalized to ribosomal S12 levels and is reported as fold increase over untreated controls (n = 4, p<0.05). **B,** Protein extracts were collected from CGNs derived from Puma+/+ and Puma−/− littermates or Bim+/+ and Bim−/− littermates 8 hours after potassium withdrawal and Puma and Bim protein levels were analyzed by western blot. Actin was included as a loading control.

**Figure 2:**
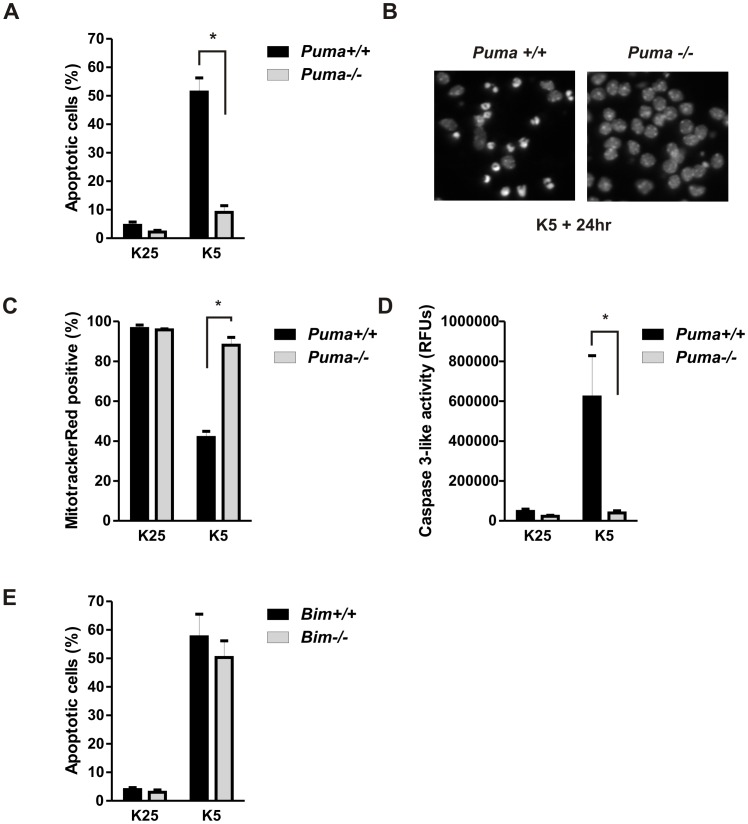
Puma is essential for potassium-withdrawal induced apoptosis in CGNs. CGNS derived Puma+/+ and Puma−/− littermates were maintained in high potassium medium (K25) or switched to low potassium medium (K5). **A,** The fraction of apoptotic cells was determined at 24 h by assessing nuclear morphology following Hoechst staining of Puma+/+ vs Puma−/− CGNs (n = 7, *p<0.01). **B,** Representative images of Hoechst stained wild type and Puma-deficient neurons 24 hours following potassium withdrawal. **C,** CGNs were stained with Mitotracker Red to assess mitochondrial membrane potential. The fraction of Mitotracker Red labeled neurons was determined 20 hours after potassium withdrawal and compared between genotypes (n = 4, *p<0.05). **D,** Cell lysates were collected 20 hours after potassium withdrawal and assayed for caspase-3-like activity. Caspase activity is reported as relative fluorescence units of cleaved caspase substrate and was compared between genotypes (n = 4,* p<0.05). **E,** CGNs derived from Bim+/+ and Bim−/− littermates were maintained in high potassium medium or switched to low potassium medium and the fraction of apoptotic cells was determined at 24 h (n = 5).

The specific BH3–only genes involved in apoptotic signaling as well as the mechanisms by which they are regulated varies depending on the cell type and death stimulus. In CGNs apoptosis induced by potassium withdrawal can be prevented by actinomycin-D or cycloheximide suggesting that *de novo* transcription is critical to the initiation of apoptosis and likely to the activation of BH3-only proteins [43]. We therefore examined expression of BH3-only genes following potassium withdrawal in CGNs using quantitative RT-PCR (Figure 1A). We found no change in transcript levels of several BH3-only family members including Noxa, Bid and Bad (data not shown), however consistent with previous reports we observed an increase in Bim and Hrk/DP5 mRNA levels [44], [45]. Interestingly, we also observed a marked increase in Puma mRNA, a relatively unstudied BH3-only member in this context (Figure 1A). Consistent with the increase in mRNA, Bim and Puma protein levels were also found to be elevated following potassium withdrawal (Figure 1B). Several studies including those from our research group have demonstrated that Puma plays a key role in regulating neuronal apoptosis in diverse injury paradigms [30], [46]–[51]. Therefore, we sought to determine whether Puma is required for potassium deprivation induced apoptotic cell death in cerebellar granule neurons. To address this we compared the level of apoptotic cells in CGNs derived from Puma-deficient and wild type littermates subjected to potassium withdrawal. We found that neurons lacking Puma exhibited a marked decrease in the number of apoptotic nuclei compared with wild type cells following potassium withdrawal (Figure 2A and 2B). One of the critical steps in the intrinsic apoptotic pathway is Bax-mediated mitochondrial depolarization and mitochondrial outer membrane permeabilization [26], [40]. Therefore we examined the role of Puma in regulating these Bax-mediated apoptotic processes. To assess mitochondrial membrane potential we stained wild type and Puma deficient neurons with the mitochondria potentiometric dye Mitotracker Red. In contrast to wildtype neurons the vast majority of Puma deficient neurons maintained the ability to uptake Mitotracker Red under low potassium conditions indicating that Puma is required for mitochondrial membrane depolarization (Figure 2C). Similarly we found that cytochrome-c was retained in the mitochondria of Puma deficient neurons indicating that Puma is required for Bax induced mitochondrial membrane permeabilization (data not shown). Furthermore, while potassium deprivation resulted in a robust induction of caspase-3-like activity in wild type neurons this was markedly reduced in Puma-deficient neurons (Figure 2D). As Bim has also been implicated in neuronal apoptosis induced by trophic factor deprivation [71], [72], we also examined the level of apoptosis in CGNs derived from Bim-null mice following potassium deprivation. In contrast to Puma-deficient CGNs we found that Bim-deficient CGNs exhibited only a modest decrease in apoptosis following potassium withdrawal as compared to wild type neurons (Figure 2E).

**Figure 3:**
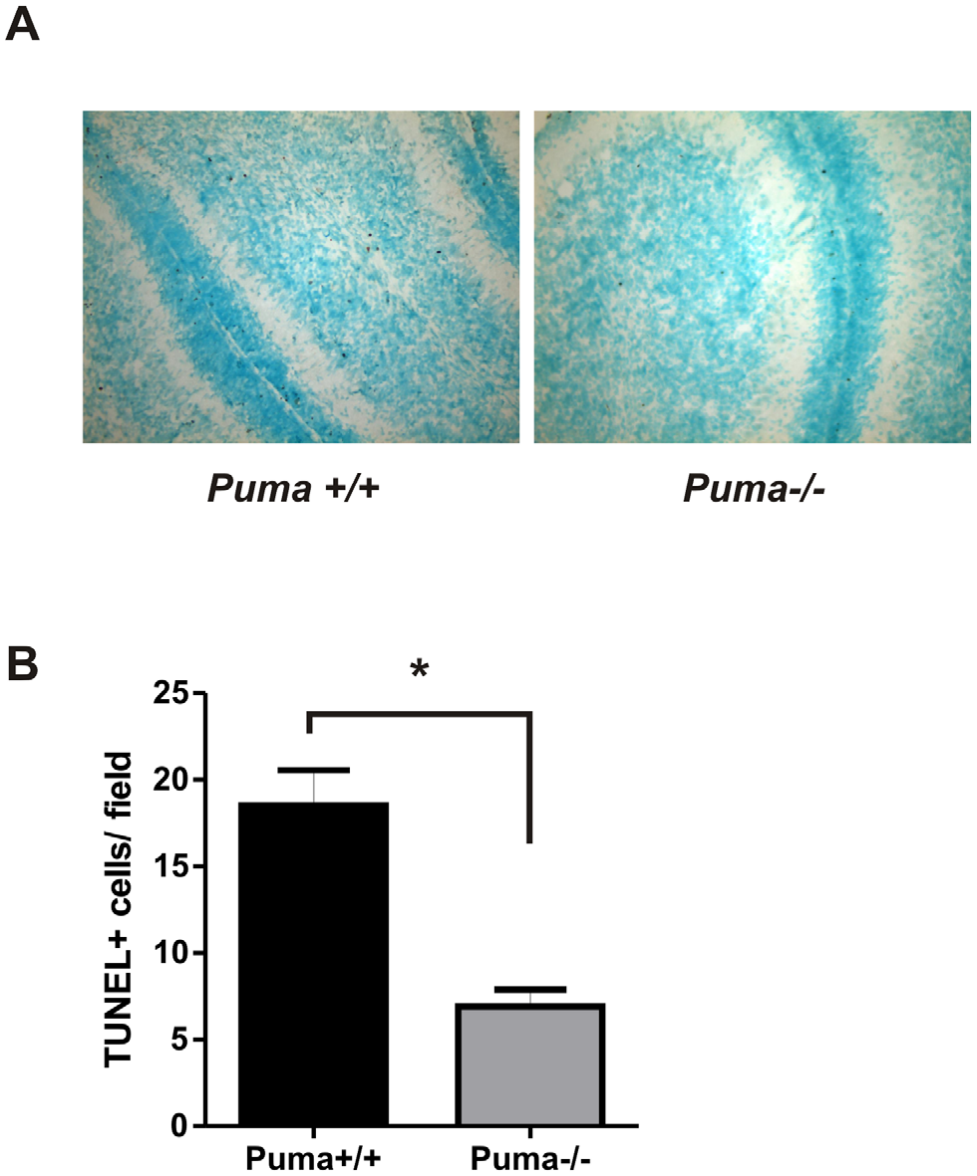
Puma contributes to CGN apoptosis during postnatal development *in vivo*. Sagittal sections from the cerebellum of postnatal day 7 Puma+/+ and Puma−/− mice were TUNEL stained to detect apoptotic cells. A, Representative images showing Tunel labeling (brown DAB+ cells) of cerebellar sections from Puma+/+ and Puma−/− mice. B, The mean number of TUNEL positive cells in the internal granule layer (IGL) per field was determined for each animal and the data is presented as the mean +/− SEM for Puma+/+ and Puma−/− mouse pups (n = 4 mice of each genotype, *p<0.05).

We next examined whether Puma contributes to cerebellar granule neuron apoptosis during postnatal development *in vivo*. As shown in Figure 3, the number of TUNEL positive cells in the cerebellar inner granule layer of post-natal day 7 Puma-deficient mice was found to be significantly reduced as compared to that in wild type mice indicating that Puma also contributes to CGN apoptosis *in vivo*. Taken together these results suggest that Puma is essential for Bax activation and apoptotic cell death induced by trophic factor deprivation in CGNs.

### JNK Activation is Required for Puma Induction During Potassium Deprivation Induced Apoptosis

**Figure 4:**
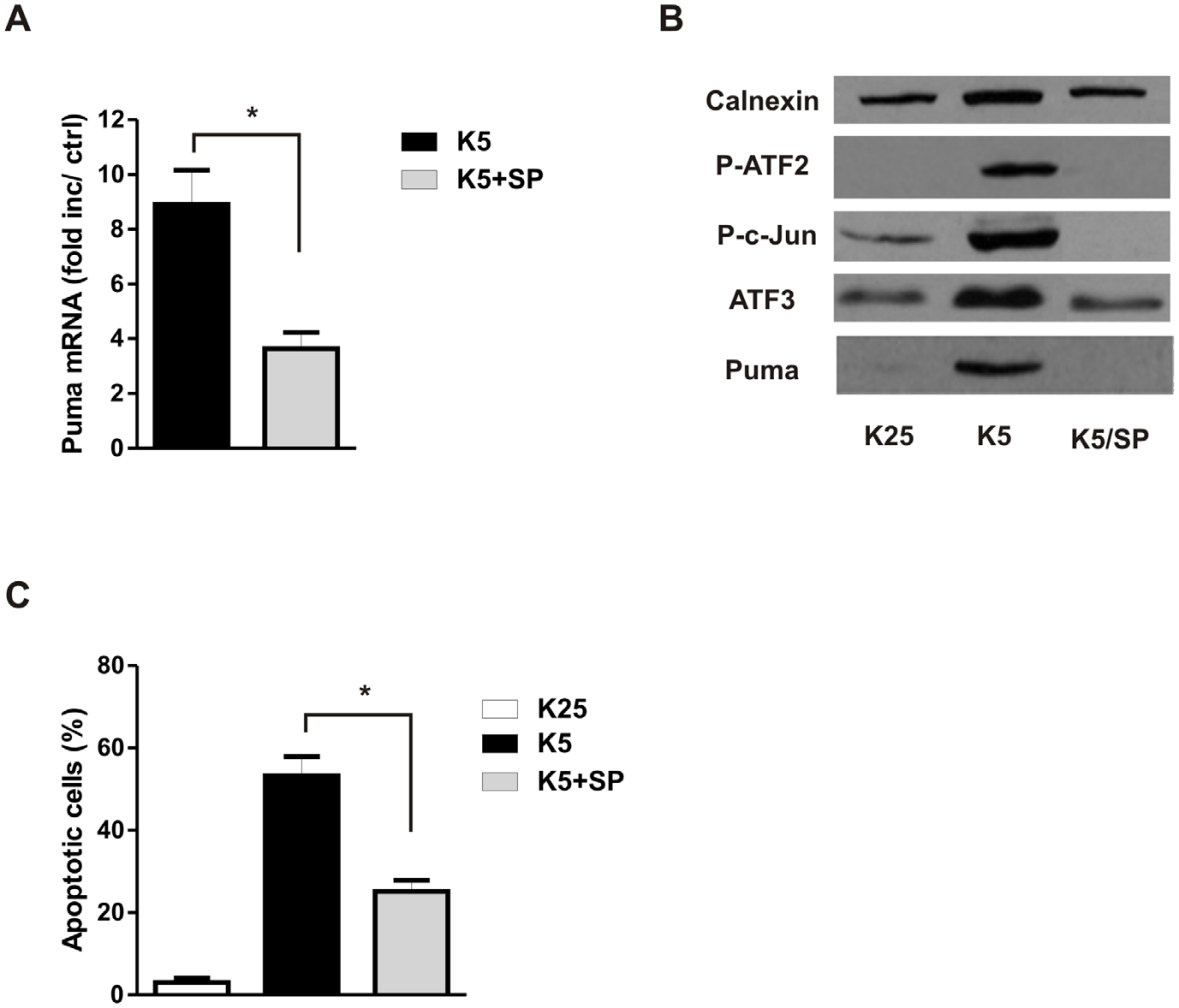
JNK is required for Puma induction and potassium withdrawal induced neuronal apoptosis. After 7 days in culture CGNs were either maintained in high potassium medium or switched to low potassium medium with or without 10 µM SP600125 (SP). **A,** Puma mRNA levels were assessed by qRT-PCR 6 hours after potassium withdrawal and are reported as fold increase over K25 controls (n = 7, *p<0.05). **B,** Protein extracts were collected 8 hours after potassium withdrawal and Phospho-ATF2, Phospho-c-Jun, ATF3 and Puma protein levels were assessed by western blot. Calnexin was included as a loading control. **C,** The fraction of apoptotic neurons was determined 24 hours after potassium withdrawal by examining nuclear morphology in Hoechst stained cells (n = 5, *p<0.05).

The c-Jun N-terminal kinase (JNK) pathway has been found to promote cell death signaling in several models of apoptosis including potassium withdrawal in CGNs [7], [36]. In light of our finding that Puma induction is required for apoptosis we examined whether JNK signaling was required for Puma induction in this paradigm. Indeed we found that the potassium deprivation induced increase in Puma mRNA levels was markedly reduced in the presence of the JNK inhibitor SP600125 (Figure 4A). Furthermore, we found that JNK inhibition also prevented the potassium withdrawal induced increase in Puma protein as well as the induction of several known JNK responsive transcription factors including ATF3, P-ATF2 and P-c-Jun (Figure 4B). Consistent with its effects on Puma expression JNK inhibition significantly decreased the level of apoptosis in potassium deprived CGNs (Figure 4C). These results suggest that JNK signaling is required for Puma induction during potassium deprivation induced neuronal apoptosis.

### Protein Kinase B/AKT Inactivation is Required for Puma Induction in Potassium Deprivation Induced Neuronal Apoptosis

**Figure 5:**
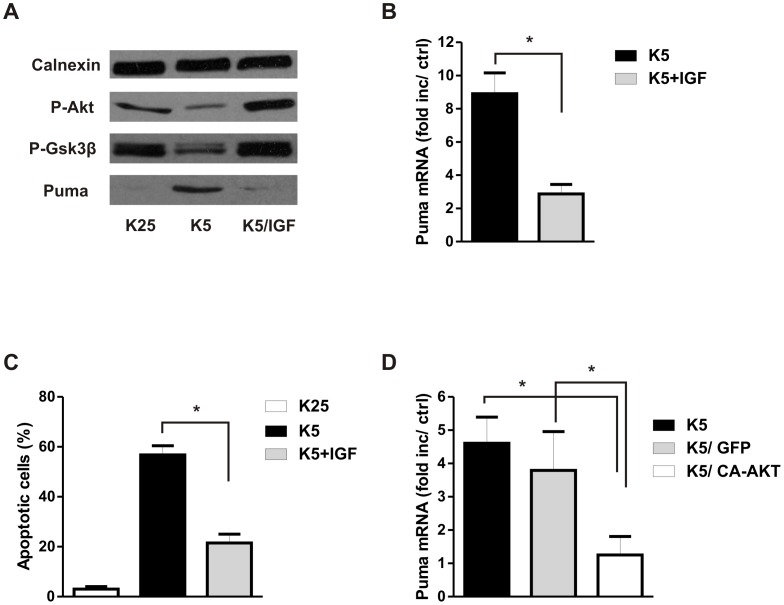
Activation of the AKT pathway inhibits potassium withdrawal induced Puma expression and neuronal apoptosis. CGNs were maintained in high potassium medium or switched to low potassium medium with or without 200 nM IGF-1. **A,** Protein extracts were collected 8 hours after potassium withdrawal and Phospho-AKT, Phospho-GSK3β and Puma protein levels were assessed by western blot. **B,** RNA was collected 6 hours after potassium withdrawal and Puma mRNA levels were quantified by qRT-PCR. Puma expression is reported as fold increase over K25 controls (n = 4,*p<0.05). **C,** Neurons were Hoechst stained 24 hours after potassium withdrawal in the presence or absence of IGF-1 (200 nM) and the fraction of apoptotic cells was determined by examining nuclear morphology (n = 4, *p<0.05). **D,** CGNs were infected with adenovirus expressing either GFP or a constitutively active form of AKT (CA-AKT) at 10 MOI and seven days later cells were switched to low potassium medium to induce apoptosis. RNA was collected 6 hours after potassium withdrawal and Puma transcript levels were quantified by qRT-PCR. Puma mRNA levels are reported as fold increase over K25 controls (n = 3, *p<0.05).

Protein kinase B (or AKT) is also known to modulate neuronal apoptosis but in contrast to the JNK pathway it does so in a pro-survival manner [6]. It has previously been demonstrated that AKT activity is decreased in trophic factor deprived neurons and that activation of the PI3K-AKT pathway is neuroprotective [22], [23]. Therefore we examined whether AKT inactivation may also be involved in the regulation of Puma expression. To address this we examined Puma induction in potassium deprived CGNs in the presence or absence of insulin-like growth factor-1(IGF-1) a known activator of the PI3K-AKT pathway [21]. As shown in Figure 5A, IGF-1 prevented the potassium withdrawal induced decrease in P-AKT levels and suppressed the increase in Puma protein. Consistent with this, IGF-1 also significantly decreased Puma mRNA induction in potassium deprived neurons (Figure 5B) and protected against apoptotic cell death (Figure 5C). IGF-1 can activate pathways in addition to AKT therefore to further examine the role of AKT we compared Puma mRNA levels in CGNs transduced with a recombinant adenovirus expressing constitutively active AKT (CA-AKT) or green fluorescent protein (GFP) as a control. As shown in Figure 5D, Puma mRNA induction by potassium deprivation was significantly reduced in CGNs expressing CA-AKT as compared to Ad-GFP infected or uninfected neurons.

**Figure 6:**
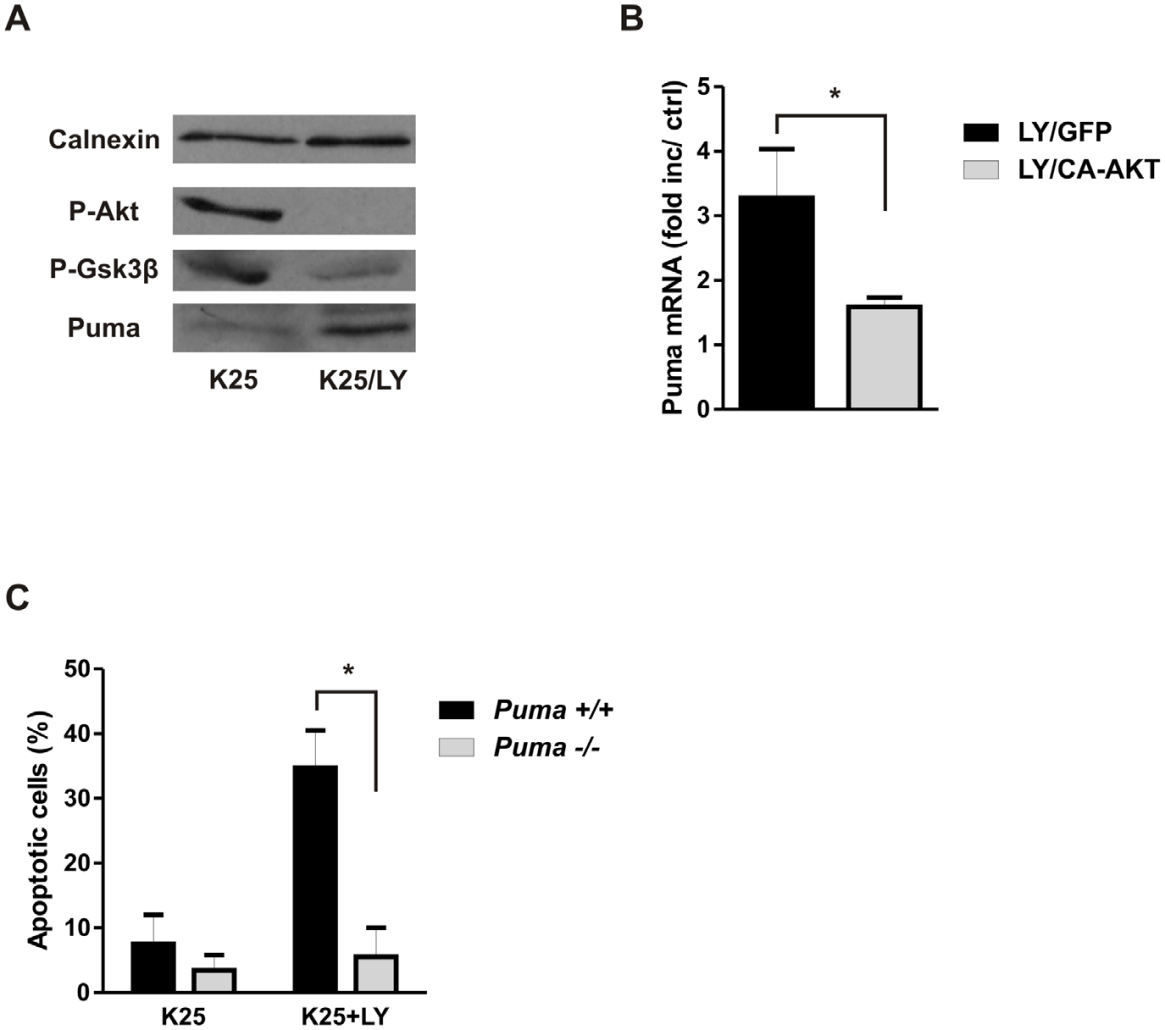
PI3K/AKT inhibition induces Puma expression and Puma-dependent apoptosis in CGNs. **A,** CGNs maintained in high potassium medium (K25) were treated with the PI3K inhibitor LY294002 (30 µM) or vehicle and protein extracts were collected after 12 hours and Phosph-AKT, Phospho-GSK3β and Puma protein levels were assessed by western blot. **B,** CGNs were infected with recombinant adenovirus expressing either CA-AKT or GFP at 10 MOI and after 7 days neurons were treated with or without LY294002 (30 µM). RNA was collected at 8 hours and Puma mRNA levels were quantified by qRT-PCR (n = 4, *p<0.05). **C,** CGNs derived from Puma+/+ and Puma−/− littermates were treated with or without 30 µM LY294002 (LY) and the fraction of apoptotic neurons was quantified after 24 hours by Hoechst staining.

Previous studies suggest that inhibition of the PI3K/AKT pathway is in itself sufficient to induce apoptosis in neurons [20], [23], [35]. Therefore we investigated whether cell death induced by AKT inactivation was mediated by Puma. To address this we examined Puma expression in CGNs treated with the PI3K inhibitor LY294002 under high (normal) potassium conditions. PI3K inhibition by LY294002 resulted in a substantial reduction in P-AKT levels and a corresponding increase in Puma protein and mRNA levels (Figures 6A and 6B). We found that the increase in Puma mRNA expression induced by LY294002 was attenuated in CGNs expressing CA-AKT suggesting that AKT inactivation is primarily responsible for the LY294002 induced Puma expression (Figure 6B). Finally, to determine whether Puma is necessary for neuronal cell death induced by PI3K-AKT inactivation we examined LY294002 induced apoptosis in CGNs derived from Puma-deficient mice and wild type littermates. As depicted in Figure 6C, LY294002 induced significant levels of apoptosis in wild type (∼35% at 24 hours) but not Puma deficient (<10%) neurons indicating that Puma is necessary for cell death induced by PI3K-AKT inactivation. Taken together these results suggest that AKT inactivation is a key determinant of Puma induction in neuronal apoptosis.

### AKT Functions Through GSK3β to Modulate Puma-mediated Apoptosis

**Figure 7:**
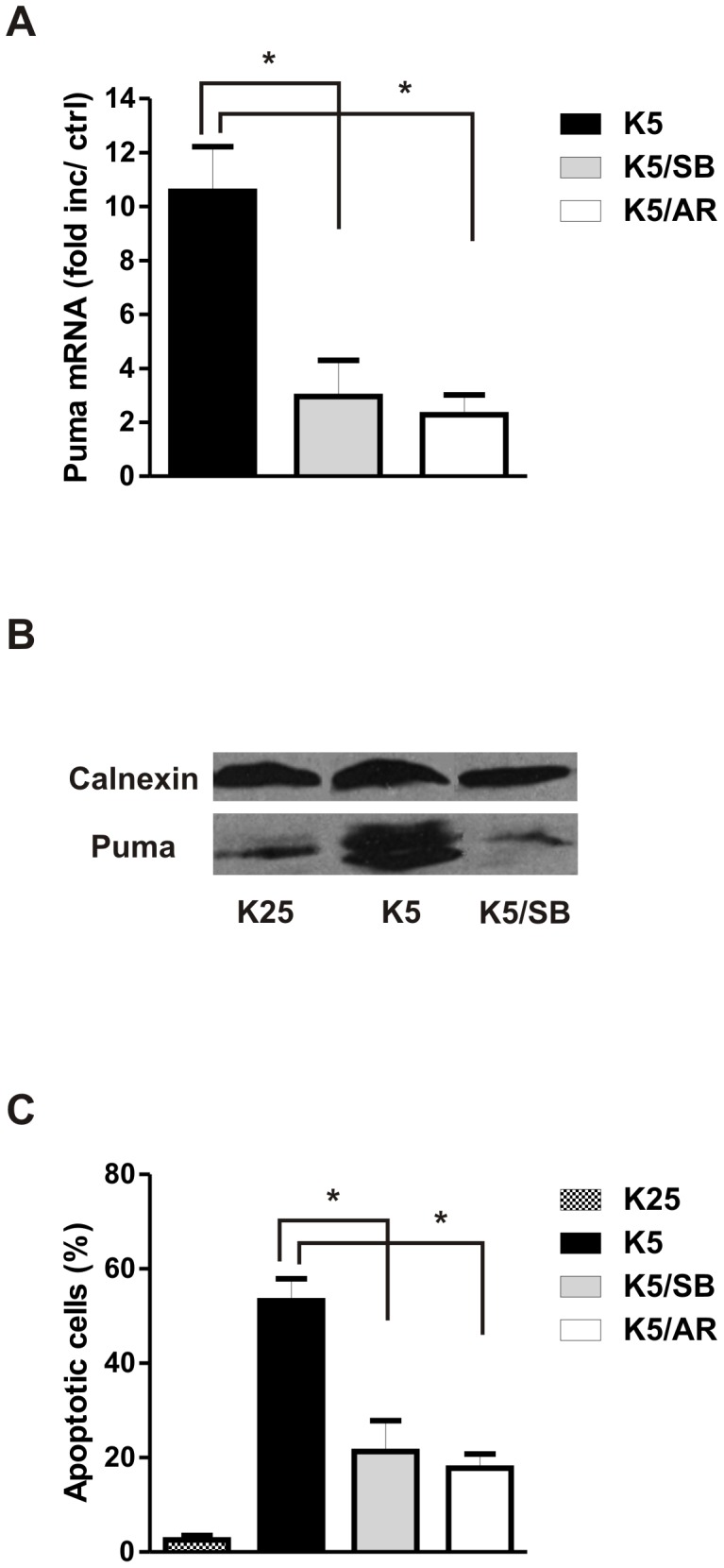
GSK3β is required for Puma induction in potassium withdrawal induced apoptosis. CGNs were switched to low potassium medium in the presence or absence of the GSK3α/β inhibitor SB415286 (SB, 30 µM) or the GSK3β specific inhibitor AR-A01 4418 (AR, 50 µM). **A,** RNA was collected six hours after potassium withdrawal and analyzed for Puma mRNA expression using qRT-PCR (n = 4, *p<0.05). **B,** Protein extracts were collected 8 hours after potassium withdrawal and Puma protein levels were analyzed by western blot. **C,** The fraction of apoptotic neurons was quantified after 24 hours by Hoechst staining (n = 3, *p<0.05).

**Figure 8:**
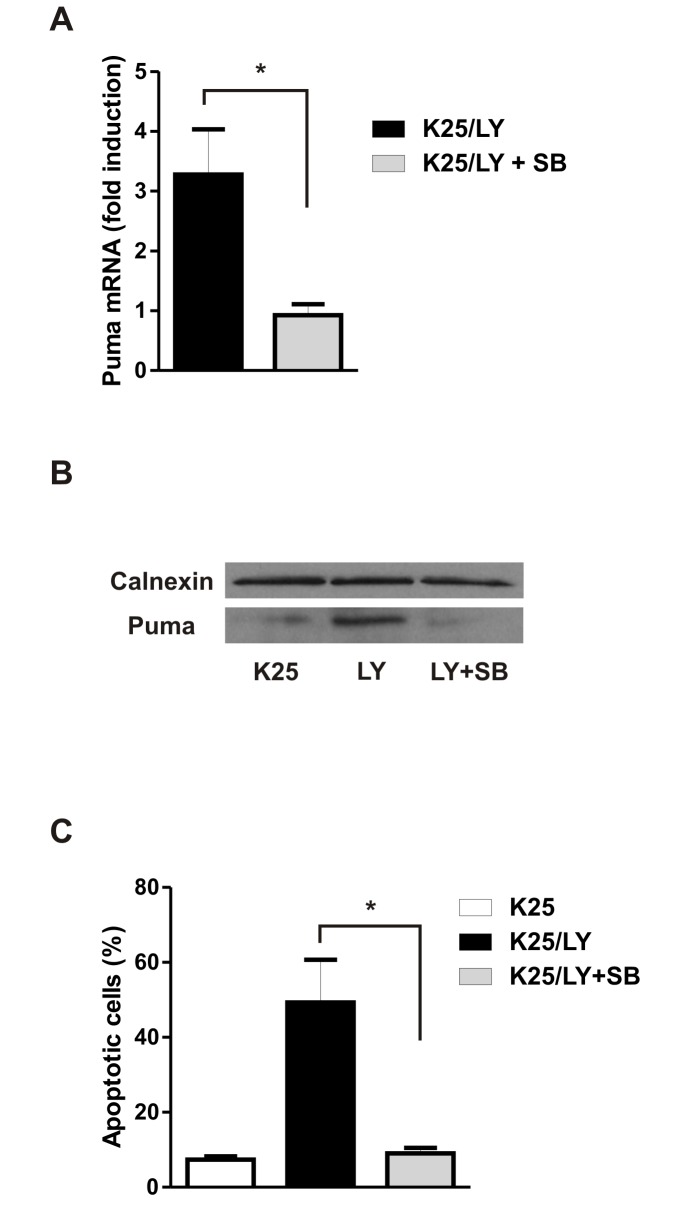
AKT inactivation induces Puma expression and neuronal apoptosis via a GSK3β-dependent mechanism. CGNs maintained for 7 days in high potassium medium were treated with or without the PI3K inhibitor LY294002 (30 µM, LY) in the presence or absence of the GSK3β inhibitor SB415286 (30 µM, SB). **A,** RNA was collected 8 hours post treatment and Puma mRNA levels were determined by qRT-PCR (n = 3,* p<0.05). **B,** Protein extracts were collected 12 hours post-treatment and Puma protein levels were analyzed by western blot. **C,** The fraction of apoptotic neurons was quantified after 24 hours by Hoechst staining (n = 3, *p<0.05).

Glycogen synthase kinase 3β (GSK3β) has been found to play a pro-apoptotic role in several models of neuronal apoptosis (reviewed in [8]) including potassium withdrawal in CGNs [52], [53]. GSK3β activity is known to be inhibited by AKT mediated serine-9 phosphorylation and inactivation of AKT results in GSK3β activation associated with serine-9 dephosphorylation [22]. Indeed we find that GSK3β serine-9 phosphorylation is decreased in potassium deprived neurons consistent with its activation, and that IGF-1 prevents this dephosphorylation/activation (Figure 5A). Similarly, we find that direct inhibition of PI3K/AKT by LY294002 is sufficient to induce GSK3β-dephosphorylation/activation (Figure 6A). Therefore, we investigated whether GSK3β activation may link AKT inactivation to Puma induction and neuronal cell death. To address this we examined Puma expression in CGNs deprived of potassium in the presence of the GSK3α/β inhibitor SB415286 or the GSK3β selective inhibitor AR-A014418 [54], [55]. As shown in Figures 7A and 7B, the induction of Puma mRNA and protein by potassium deprivation was significantly reduced by the GSK3β inhibitors. GSK3β inhibition also significantly reduced the level of apoptosis induced by potassium deprivation (Figure 7C). We next examined the role of GSK3β in Puma expression and cell death induced by LY294002 mediated PI3K/AKT inactivation. Inhibition of GSK3β by the SB415286 compound abolished LY294002 induced Puma mRNA and protein (Figures 8A and 8B) as well as LY induced apoptosis (Figure 8C). Taken together these results suggest that AKT inactivation triggers Puma induction and neuronal apoptosis via a GSK3β-dependent mechanism.

### The JNK and AKT-GSK3β Pathways Converge to Regulate FoxO3a Mediated Transcriptional Induction of Puma

**Figure 9:**
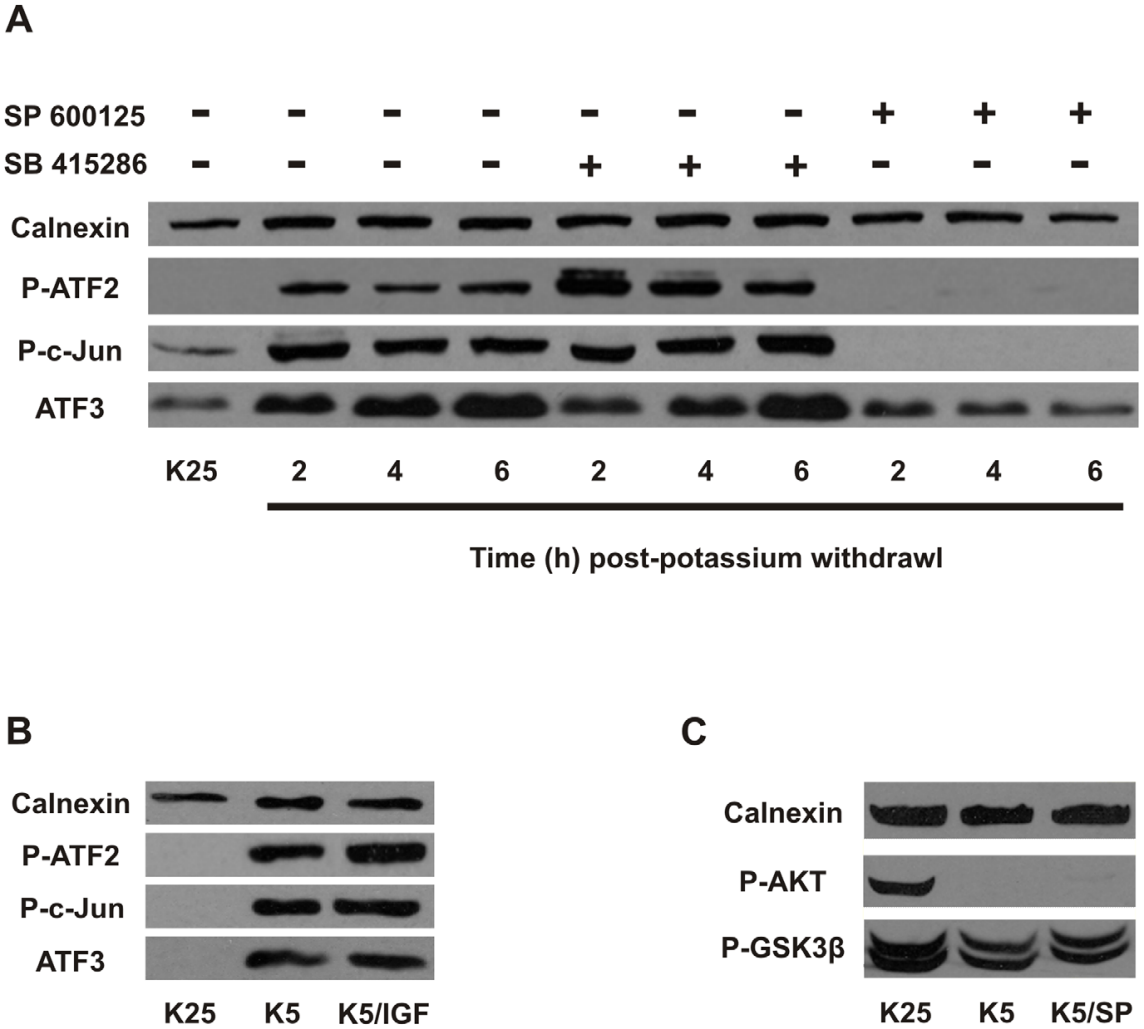
The JNK and AKT/GSK3β pathways signal independently during potassium withdrawal in CGNs. **A,** CGNs were maintained in high potassium medium (K25) or subjected to potassium withdrawal (K5) in the presence or absence of the JNK inhibitor SP600125 (10 µM) or the GSK3β inhibitor SB415286 (30 µM). Protein extracts were collected at 2, 4 and 6 hours and Phospho-ATF2, Phospho-c-Jun and ATF3 levels were analyzed by western blot. **B,** CGNs were subjected to potassium withdrawal in the presence or absence of 200 nM IGF-1. Protein extracts were collected after 6 hours and were analyzed for Phospho-ATF2, Phospho-c-Jun and ATF3 protein levels by western blot. **C,** CGNs were subjected to potassium withdrawal in the presence or absence of 10 µM SP600125 (SP) and protein extracts were collected after 6 hours and analyzed by western blot for Phospho-AKT and Phospho-GSK3β levels.

Having established a requirement for both the JNK and AKT/GSK3β pathways in Puma induction we next examined whether these signaling pathways were co-dependent or signaling independently of one another. We found that inhibition of GSK3 did not affect the potassium withdrawal induced upregulation of downstream JNK targets including P-c-Jun, P-ATF2 and ATF3 implying that JNK signaling is not dependent on GSK3β activity (Figure 9A). Furthermore, JNK downstream targets are not affected by AKT signaling independently of GSK3β as their induction is not affected by AKT activation by IGF-1 (Figure 9B). Finally, we find that AKT and GSK3β phosphorylation levels are not affected by SP600125 mediated JNK inhibition suggesting that JNK is not indirectly modulating the activity of the AKT/GSK3β pathway (Figure 9C). Taken together these results suggest that the JNK and AKT/GSK3β pathways function independently of one another during potassium withdrawal in CGNs.

**Figure 10:**
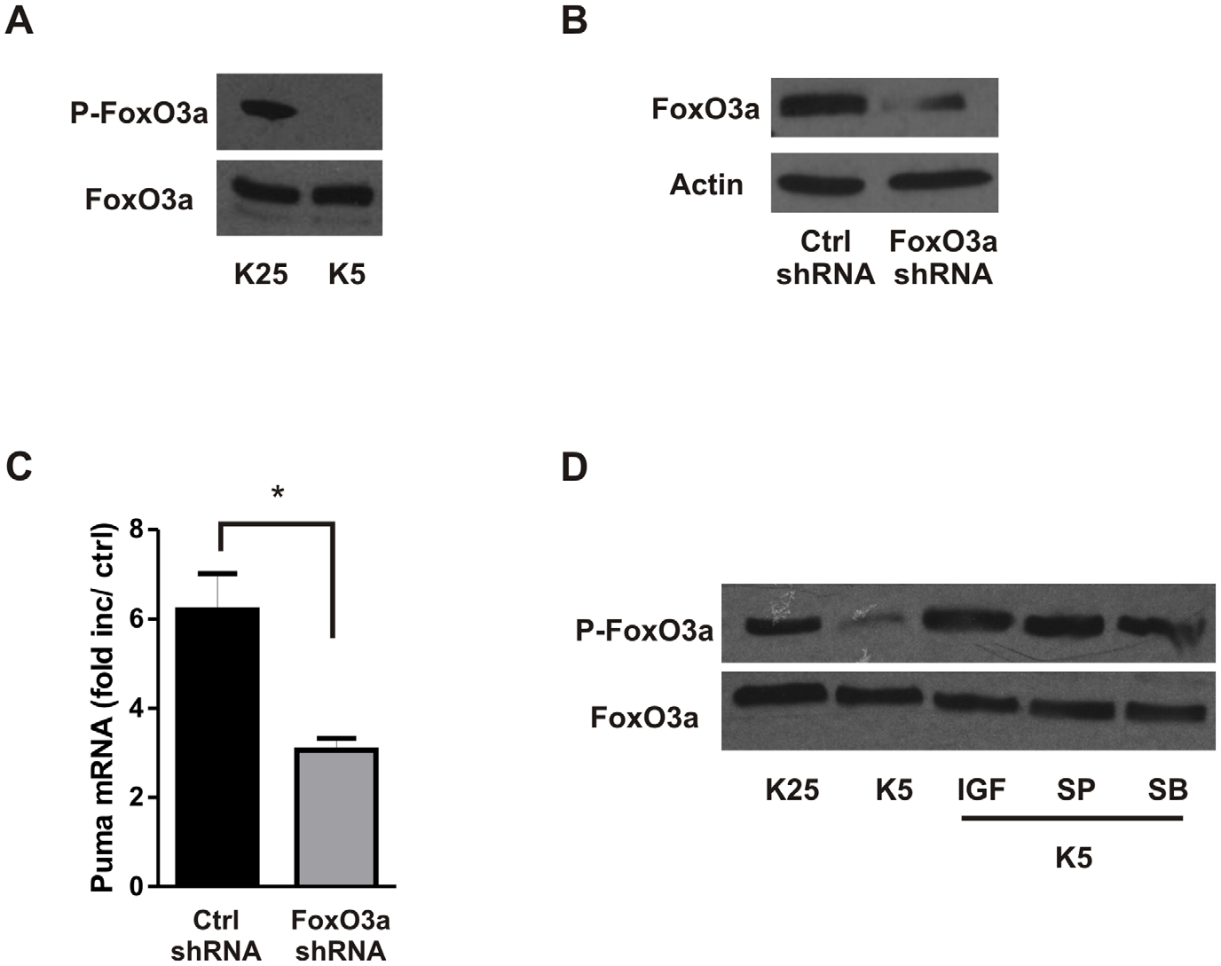
FoxO3a regulates Puma induction in potassium deprived CGNs. **A,** CGNs were maintained in high potassium (K25) or subjected to potassium withdrawal (K5). Protein extracts were collected at 8 hours and Phospho(Thr32)-FoxO3a and total FoxO3a levels were analyzed by western blot. **B,** CGNs were transduced with lentivirus (1 MOI) expressing shRNA directed against FoxO3a or a non-targeting control shRNA and FoxO3a protein levels were analyzed by western blot at 7 days post-infection. **C,** Puma mRNA levels were measured by qRT-PCR following 8 hours of potassium deprivation in CGNs transduced with FoxO3a-shRNA or non-targeting control shRNA (n = 3; *p<0.05). **D,** Phospho(Thr32)-FoxO3a and total FoxO3a protein was analyzed by western blot in CGNs maintained in high potassium (K25) or subjected to potassium deprivation for 8 hours in the presence or absence of either IGF-1 (200 nM), SP600125 (10 µM) or SB415286 (30 µM).

The transcription factor FoxO3a is known to be inactivated via phosphorylation by AKT [78]. Furthermore, FoxO3a has been implicated in the regulation of Puma expression in growth factor withdrawal induced apoptosis of lymphoid cells [80]. Therefore, we examined whether FoxO3a is required for Puma induction in potassium deprivation induced apoptosis of CGNs. Consistent with the decrease in AKT activity we found that FoxO3a (Thr-32) phosphorylation was reduced in CGNs following potassium deprivation (Figure 10A). To determine whether FoxO3a is required for Puma induction in this paradigm, we transduced CGNs with lentivirus expressing shRNA targeting FoxO3a or a non-targeting shRNA as a control. As shown in Figures 10B and 10C, FoxO3a knockdown resulted in a significant decrease in Puma mRNA induction in response to potassium withdrawal suggesting that FoxO3a contributes to Puma induction in trophic factor deprived CGNs. We next examined whether AKT, GSK3β and JNK signaling affected potassium deprivation induced FoxO3a dephosphorylation/activation. Consistent with its ability to promote AKT activation, IGF-1 suppressed the potassium deprivation induced dephosphorylation of FoxO3a. Interestingly, however, we found that inhibition of either JNK or GSK3 also attenuated potassium deprivation induced FoxO3a dephosphorylation/activation (Figure 10D). These results suggest that JNK and GSK3β signaling are also required for potassium deprivation induced FoxO3a activation although the mechanism remains unclear.

In summary, we have established a novel link between kinase pathways and the transcriptional activation of the Bcl-2 family protein Puma that is critical for the execution of neuronal apoptosis. We propose a model in which the JNK and AKT/GSK3β pathways are activated independently and converge to regulate transcription factors including FoxO3a that mediate transcriptional induction of Puma which in turn promotes Bax activation and neuronal cell death.

## Discussion

Apoptosis has been implicated in the progression of acute and chronic neurodegenerative conditions such as stroke, spinal cord injury, Alzheimer’s disease, Parkinson’s disease and Huntington’s disease. Several kinases have been implicated in the regulation of neuronal apoptosis including AKT, GSK3 and JNK family kinases. The AKT pathway has been found to promote cell survival in many neuronal cell types [22], [35], [56]–[59] while inhibition of AKT signaling has been shown to promote neuronal cell death [20], [23], [35]. In contrast, the GSK3β and JNK family kinases are known to function to promote cell death in several types of neurons and inhibition or knockdown of these kinases protects neurons from a variety of apoptotic stimuli [11], [14], [60]–[64]. While it is well established that these kinases play a key role in determining neuronal survival the mechanisms by which they regulate the apoptotic machinery remains unclear. Importantly, in the present study we have demonstrated that the AKT, GSK3β and JNK signaling pathways converge to regulate the transcriptional induction of the pro-apoptotic Bcl-2 family member Puma. Furthermore we demonstrate that induction of Puma by these kinase pathways is a critical determinant of apoptosis in cerebellar granule neurons both *in vitro* and *in vivo*.

The Bcl-2 family proteins are critical mediators of apoptosis and several studies have demonstrated that the multi-domain pro-apoptotic member Bax is essential for the execution of apoptosis in diverse neuronal death paradigms [26]–[30], [65], [66]. It is now recognized that the BH3-only subfamily of Bcl-2 proteins play a key role in activating Bax in response to apoptotic stimuli making them likely candidates for kinase mediated regulation [67]. The BH3-only family consists of multiple members and indeed several of these have been shown to be affected by AKT and JNK signaling. For example, AKT has been reported to phosphorylate Bad resulting in its sequestration by protein 14-3-3 and inhibiting its ability to induce apoptosis [68], [69]. Similar to our results with Puma, it has been reported that AKT upregulation by IGF-1 can suppress the transcriptional induction of Bim in potassium deprived CGNs [70]. Furthermore, it has been shown that JNK inhibition can block transcriptional induction of the BH3-only members Bim and Hrk/DP5 in trophic factor deprived neurons [44], [71], [72]. The role of Hrk/DP5 in trophic factor deprivation induced neuronal apoptosis appears to be neuronal subtype dependent as apoptosis is not reduced in Hrk/DP5-deficient CGNs subjected to potassium deprivation, but is partially reduced in superior cervical ganglia cells following nerve growth factor withdrawal [73]. Similarly, it has previously been reported that trophic factor deprivation induced apoptotic cell death is significantly reduced in Bim-deficient neurons [71]. However, we have found that potassium deprivation induced apoptosis is only modestly reduced in Bim-deficient CGNs. On the other hand we have determined that Puma plays a major role in regulating trophic factor deprivation induced apoptosis in CGNS both *in vitro* and *in vivo*. Furthermore, Puma-deficient neurons have been shown to be remarkably resistant to the induction of apoptosis by diverse stimuli including DNA damage, oxidative stress, ER stress/dysfunction, and proteasome inhibition [30], [46], [47], [50]. In addition, Puma-deletion has been shown to be neuroprotective in mouse models of severe *status epilepticus* and Amyotrophic Lateral Sclerosis [49], [51].

We have determined that inhibition of either JNK or GSK3β markedly reduces Puma induction and cell death suggesting that simultaneous activation of both pathways is required for Puma induction. Furthermore, our results suggest that these pathways are functioning independently and converge to regulate Puma transcription. Specifically we have determined that suppression of the AKT/GSK3β pathway by either IGF-1 mediated AKT activation or pharmacological inhibition of GSK3β does not affect the induction of JNK targets including P-c-Jun, P-ATF2 or ATF3. Similarly, we find that inhibition of JNK does not affect AKT activity as it does not appear to influence AKT mediated GSK3β (ser-9) phosphorylation. However, we cannot rule out the possibility that JNK could indirectly modulate GSK3β activity independently of AKT. Interestingly, we found that prolonged inactivation of the PI3K-AKT pathway by LY294002 was sufficient to induce Puma expression and neuronal cell death. However, we found that cell death induced by LY294002 was inhibited by the JNK inhibitor SP600125 (data not shown) suggesting that basal levels of JNK activity may be contributing to Puma induction in this context. This would be consistent with the lower levels of Puma induction and cell death observed following LY294002 mediated PI3K/AKT inactivation as compared with potassium withdrawal. Our finding that activation of both the AKT/GSK3β and JNK pathways is required to control Puma induction suggests a signaling cascade which has a built-in safety mechanism to prevent spontaneous neuronal apoptosis.

The activation of Puma mRNA induction provides the convergence point of these kinase signaling pathways, however, the exact mechanism by which they converge on Puma induction remains to be determined. As Puma is regulated at the transcriptional level it seems logical that these kinases alter the activity of transcriptional repressors or activators which in turn control Puma expression. Puma was originally identified as a target gene of the transcription factor p53, and indeed our laboratory, as well as others have demonstrated that Puma is an essential pro-apoptotic factor in p53-mediated neuronal apoptosis [50], [74], [75]. However, Puma has been shown in many cases to be induced independently of p53 (reviewed in [76]), and it is unlikely that p53 contributes to Puma induction in this model as it has previously been demonstrated that p53 is not required for potassium withdrawal induced apoptosis in CGNs [77]. As such, we expected that other transcription factors, downstream of the AKT/GSK3β and JNK pathways, would be responsible for Puma upregulation following potassium deprivation in CGNs. Previous studies have implicated the transcription factor FoxO3a in trophic factor deprivation induced neuronal cell death [70], [79]. Importantly, we demonstrate that FoxO3a promotes neuronal apoptosis through the transcriptional induction of Puma. Similar to our results it has previously been reported that FoxO3a can activate Puma transcription and apoptosis in cytokine-deprived lymphoid cells [80]. The nuclear localization and transcriptional activity of FoxO3a is negatively regulated by AKT-mediated phosphorylation. Consistent with this we found that IGF-1 prevented the potassium deprivation induced decrease in AKT activity, FoxO3a dephosphorylation and attenuated Puma induction. Interestingly, we found that inhibition of either JNK or GSK3β also inhibited FoxO3a dephosphorylation/activation. These results were surprising given that GSK3β is activated downstream of AKT and that JNK signaling does not appear to affect AKT activity in this context (Figure 9C). This suggests that JNK and GSK3β can regulate FoxO3a phosphorylation by an indirect mechanism or via an AKT-independent mechanism perhaps by regulating the activity of a phosphatase involved in FoxO3a dephosphorylation.

Although JNK and GSK3β were found to affect FoxO3a activation we cannot rule out the possibility that they may also regulate other transcription factors involved in Puma induction. A candidate factor downstream of GSK3β is nuclear factor of activated T cells (NFAT) which has been shown to be phosphorylated by GSK3β resulting in its export from the nucleus and promotion of survival in CGNs [81], [82]. In this case NFAT may act as a repressor of Puma transcription which is removed upon GSK3β activation. Similarly, beta-catenin may be acting to suppress Puma induction until inactivated by GSK3β. Phosphorylation of beta-catenin by GSK3β causes its translocation out of the nucleus and targets it for degradation and inhibition of this phosphorylation event has been associated with neuronal survival [83], [84]. Finally, there are several downstream targets of the JNK pathway which could control Puma expression following JNK activation, these include c-Jun, activating transcription factor 2 (ATF2) and activating transcription factor 3 (ATF3). A main downstream target of JNK, c-Jun has been found to be upregulated in trophic factor deprived neurons and ectopic expression of dominant negative c-Jun was found to protect against cell death [64], [85]–[87]. The JNK regulated transcription factors ATF2 and ATF3 are also induced in response to potassium deprivation and it has been reported that knockdown or inhibition of these factors can protect neurons against apoptosis [88], [89]. It is noteworthy that the Puma promoter contains putative AP1 binding sites which are the known target sequence for all three of these transcription factors, suggesting a potential role for these factors in Puma induction. Interestingly, a recent study implicated c-Jun in the regulation of Puma expression in fatty acid induced apoptosis of hepatocytes [90], although the AP-1 binding site identified in this study does not appear to be conserved. While these transcription factors have been implicated in neuronal apoptosis it is unclear whether or not they play a role in Puma upregulation in this context and is currently under investigation.

In summary, we have delineated a key pathway involved in the regulation of apoptosis induced by potassium deprivation in CGNs. Cell death in this paradigm results from the loss of activity dependent survival signals which is believed to mimic aspects of synaptic dysfunction common to many neuronal injury and neurodegenerative conditions. Therefore, in future studies it will be important to investigate the role of this pathway in *in vivo* models of neuronal injury and neurodegenerative diseases and to explore the therapeutic potential of targeting this pathway.
